# Dietary Omega-3 Polyunsaturated Fatty Acid Deprivation Does Not Alter Seizure Thresholds but May Prevent the Anti-seizure Effects of Injected Docosahexaenoic Acid in Rats

**DOI:** 10.3389/fneur.2018.01188

**Published:** 2019-02-05

**Authors:** Ameer Y. Taha, Marc-Olivier Trepanier, Flaviu A. Coibanu, Anjali Saxena, Melanie A. Jeffrey, Nadeen M. Y. Taha, W. McIntyre Burnham, Richard P. Bazinet

**Affiliations:** ^1^Department of Food Science and Technology, College of Agriculture and Environmental Sciences, University of California, Davis, Davis, CA, United States; ^2^EpLink, the Epilepsy Research Program of the Ontario Brain Institute, Toronto, ON, Canada; ^3^Department of Nutritional Sciences, Faculty of Medicine, University of Toronto, Toronto, ON, Canada; ^4^Department of Pharmacology and Toxicology, Faculty of Medicine, University of Toronto, Toronto, ON, Canada

**Keywords:** after-discharge seizure threshold, amygdala, pentylenetetrazol, omega-3 deficiency, DHA

## Abstract

**Background:** Brain concentrations of omega-3 docosahexaenoic acid (DHA, 22:6n-3) have been reported to positively correlate with seizure thresholds in rodent seizure models. It is not known whether brain DHA depletion, achieved by chronic dietary omega-3 polyunsaturated fatty acid (PUFA) deficiency, lowers seizure thresholds in rats.

**Objective:** The present study tested the hypothesis that lowering brain DHA concentration with chronic dietary n-3 PUFA deprivation in rats will reduce seizure thresholds, and that compared to injected oleic acid (OA), injected DHA will raise seizure thresholds in rats maintained on n-3 PUFA adequate and deficient diets.

**Methods:** Rats (60 days old) were surgically implanted with electrodes in the amygdala, and subsequently randomized to the AIN-93G diet containing adequate levels of n-3 PUFA derived from soybean oil or an n-3 PUFA-deficient diet derived from coconut and safflower oil. The rats were maintained on the diets for 37 weeks. Afterdischarge seizure thresholds (ADTs) were measured every 4–6 weeks by electrically stimulating the amygdala. Between weeks 35 and 37, ADTs were assessed within 1 h of subcutaneous OA or DHA injection (600 mg/kg). Seizure thresholds were also measured in a parallel group of non-implanted rats subjected to the maximal pentylenetetrazol (PTZ, 110 mg/kg) seizure test. PUFA composition was measured in the pyriform-amygdala complex of another group of non-implanted rats sacrificed at 16 and 32 weeks.

**Results:** Dietary n-3 PUFA deprivation did not significantly alter amygdaloid seizure thresholds or latency to PTZ-induced seizures. Acute injection of OA did not alter amygdaloid ADTs of rats on the n-3 PUFA adequate or deficient diets, whereas acute injection of DHA significantly increased amygdaloid ADTs in rats on the n-3 PUFA adequate control diet as compared to rats on the n-3 PUFA deficient diet (*P* < 0.05). Pyriform-amygdala DHA percent composition did not significantly differ between the groups, while n-6 docosapentaenoic acid, a marker of n-3 PUFA deficiency, was significantly increased by 2.9-fold at 32 weeks.

**Conclusion:** Chronic dietary n-3 PUFA deficiency does not alter seizure thresholds in rats, but may prevent the anti-seizure effects of DHA.

## Introduction

Epilepsy is a progressive neurological disorder characterized by self-sustained periods of neuronal hyperexcitability (1, 2). Approximately one third of people with epilepsy have uncontrolled and persistent seizures despite being treated with anti-seizure medications (3). These individuals are particularly vulnerable to seizure-related psychiatric co-morbidities such as depression and anxiety, and sudden unexplained death in epilepsy (SUDEP) (4–6).

The main problem in people with epilepsy is that they have a low seizure threshold in one or more parts of the brain (1, 7). While mutations in several genes (e.g., sodium channel subunit mutations) may underlie epileptic seizures (8), environmental factors such as light or sound intensity may play a role in provoking a seizure episode in seizure-prone individuals (9, 10). Understanding factors that lower seizure thresholds may help inform on strategies that enable better seizure control in people with drug-resistant epilepsy.

Dietary lipids may also play a role in regulating seizure thresholds in epileptic patients. In particular, omega-3 polyunsaturated fatty acids (n-3 PUFAs) derived from plants (11) or seafood (12), were reported to raise seizure thresholds in rodents (13–17). The main n-3 PUFA found in the brain is docosahexaenoic acid (DHA, 22:6n-3). DHA regulates multiple processes within the brain, including gene transcription, neurotransmission, and the production of anti-inflammatory lipid mediators involved in resolving neuroinflammation (18–22).

DHA can be obtained preformed from the diet, or through liver elongation and desaturation of dietary alpha-linolenic acid (ALA, 18:3n-3) (23). ALA is thought to compete for elongation-desaturation with omega-6 linoleic acid (LA, 18:2n-6), which can be elongated-desaturated into arachidonic acid (AA, 20:4n-6) and docosapentaenoic acid (22:5n-6; DPA n-6) (24). Rats chronically fed an n-3 PUFA deficient diet show significant reductions in brain DHA concentration and increases in n-6 DPA (but not AA) concentration (25).

Mice fed an n-3 PUFA deficient diet for 30–34 days were reported to have greater susceptibility to magnesium-dependent audiogenic seizures than mice fed an n-3 PUFA adequate diet (15, 26). Consistent with these rodent studies, one epidemiological study reported a higher incidence of seizures in children born to mothers consuming low (117 mg/day) or high (817 mg/day) long-chain n-3 PUFAs during pregnancy as compared to children of mothers consuming intermediate levels of n-3 PUFAs (400–600 mg/day) (27). Another study reported reduced seizure incidence in infants born to mothers who received 800 mg/day of DHA during the second and third trimesters of pregnancy, as compared to mothers not supplemented with DHA (i.e., given a vegetable oil placebo) (28). Collectively, these studies suggest that low intake of n-3 PUFAs may reduce seizure threshold and increase the risk of seizure occurrence.

The present study tested the hypothesis that chronic dietary n-3 PUFA deprivation will lower seizure thresholds in the amygdala, a focus involved in the etiology of drug-resistant complex-partial seizures (29, 30). Seizure thresholds were measured over a period of 9 months, because we expected brain DHA levels to decrease within several months of initiating the n-PUFA deficient diets, due to the 4–12 weeks half-life of DHA in the adult rat brain (31–33). Amygdaloid seizure thresholds were also measured following acute oleic acid (OA, 18:1n-9) or DHA injection to n-3 PUFA adequate and deficient rats, to test whether seizure thresholds would increase by DHA administration. The present study also tested the effects of n-3 PUFA deficiency in the pentyleneterazol (PTZ) model of generalized tonic-clonic seizures in rats (34). Brain DHA and n-6 DPA composition was measured in a parallel group of non-seizure tested rats.

## Materials and Methods

### Subjects and Treatments

Experimental procedures followed the Canadian Council on Animal Care guidelines, and were approved by the Animal Care Committee of the Faculty of Medicine of the University of Toronto.

Male Wistar rats (Charles River, La Prairie, QC, Canada), aged 53–60 days, were housed individually in transparent plastic cages with corn-cob bedding in a 12 h light-dark cycle vivarium maintained at 21°C. Food (Teklad Global, 2018 18% Protein Rodent Diet) and water were available *ad libitum*. All subjects were handled for a period of 7 days, following arrival from the breeding farm.

**Figure 1:**
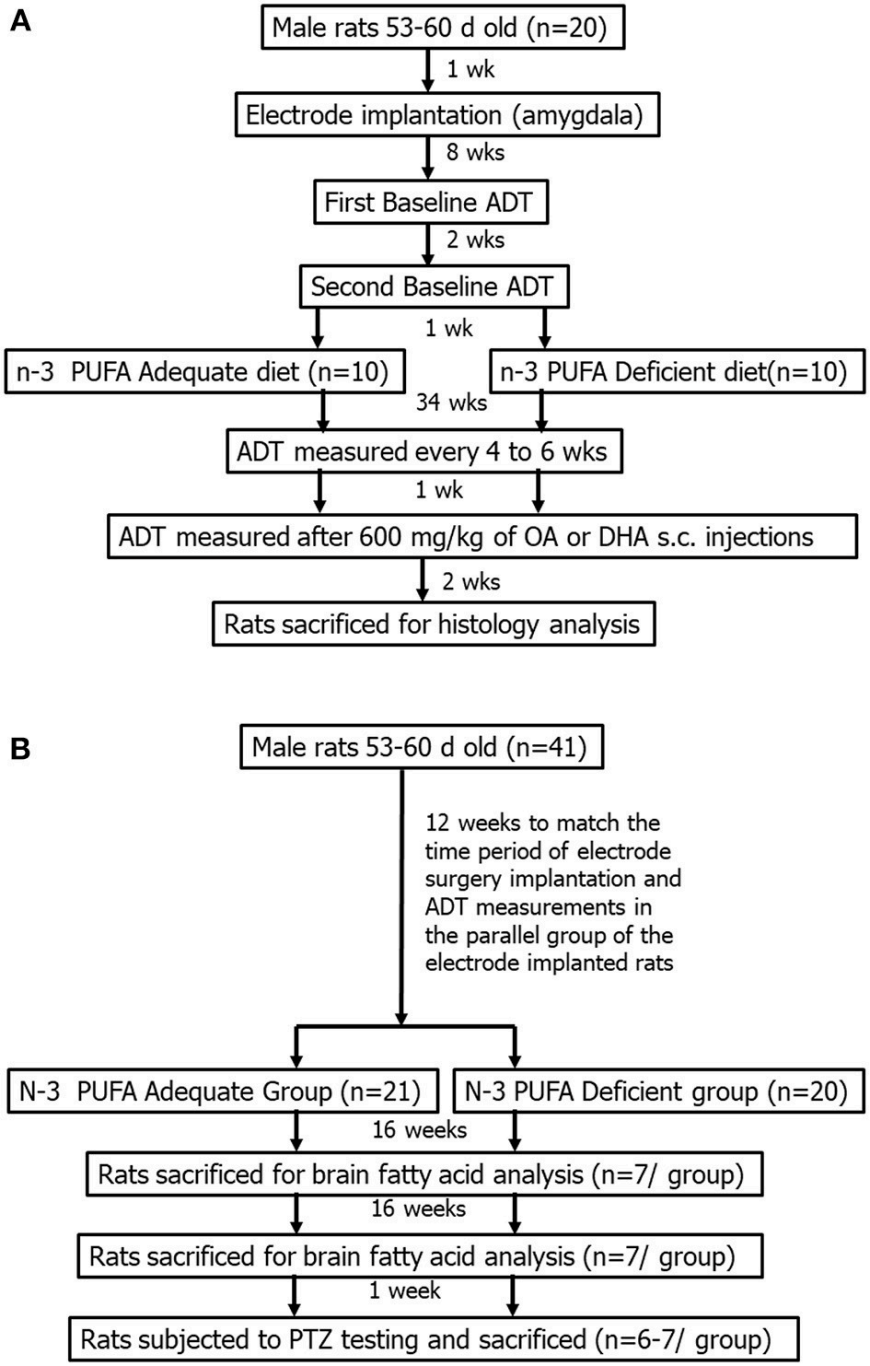
General experimental design for implanted **(A)** and non-implanted **(B)** rats. The amygdala-implanted rats **(B)** were randomized to an n-3 PUFA adequate or deficient diet for a total of 37 weeks. They were subjected to repeated ADT measurements, once every 4–6 weeks, for 34 weeks. ADTs were measured between weeks 35–37, following i.p., injection with 600 mg/kg of oleic acid (OA) or docosahexaenoic acid (DHA). The non-implanted rats **(B)** were randomized to the same diets for 33 weeks. Two-thirds of the animals were sacrificed following 16 and 32 weeks of dietary treatment, and one-third was subjected to pentylenetetrazol-induced seizures after 33 weeks of dietary treatment.

Two parallel experiments were then initiated as outlined in Figures 1A,B and described in detail below. The first experiment involved electrode implantations into the basolateral amygdala of 20 rats, followed by repeated seizure threshold measurements over a 34 week period during which the subjects were fed an n-3 PUFA adequate or deficient diet (Figure 1A). These rats were also treated with OA or DHA to test their effects on seizure threshold under the two dietary conditions. The second experiment involved a parallel group of non-implanted rats (*n* = 42) that were randomized to the n-3 PUFA adequate and deficient diets. These subjects were sacrificed after 16 or 32 weeks of dietary treatment to measure the effects of diet on brain DHA levels, or subjected to PTZ seizure testing after 33 weeks of treatment (Figure 1B).

### Procedure for Electrode Implantation

Twenty rats were surgically implanted with stainless steel bipolar electrodes (MS303/1, Plastics One, Roanoke, VA, USA) aimed at the right basolateral amygdala (*n* = 20), under isoflorane anesthesia. The amygdala coordinates were as follows: anterior-posterior, −2.8; medial-lateral (bregma), 4.6 (bregma); and dorsal-ventral, −8.6 (skull surface at bregma). The incisor bar was set and maintained in the horizontal position by aligning bregma and lambda to the horizontal plane. The electrodes were fixed to the skull with 3–4 stainless steel anchor screws and acrylic dental cement (Nuweld, LD, Caulk). All subjects were subcutaneously injected with buprenorphine analgesic (0.05 mg/kg) and physiological saline (1 ml/kg) for rehydration following surgery.

### Afterdischarge Threshold (ADT) Measurements

Baseline afterdischarge thresholds (ADTs) in the amygdala were measured 8 weeks following surgery, using the ascending series method (Figure 1A). Subjects were placed in a corn-bedded open field chamber and connected to a Grass model S-88 stimulator (Grass Instruments, Quincy, MA, USA), which delivered pulses through the recording electrode. The subjects received a one-second train of stimulation pulses at a frequency of 60 Hz, composed of a 1 ms positive and 1 ms negative phase separated by 0.5 ms. The initial stimulus intensity was 40 μA. The current was increased in steps of 20 μA up to 400 μA, and then in steps of 40 μA from 400 μA onwards, until an afterdischarge was evoked. The interval between stimulations was 5 min. The same electrodes were used for stimulating and recording focal electroencephalographic (EEG) activity.

Baseline ADT was measured again, 2 weeks later, after which, the animals were randomized to the n-3 PUFA adequate or deficient diets (see next section). ADTs were measured once every 4–6 weeks thereafter for 34 weeks. The second baseline ADT measurement was used as the reference point of comparison for assessing subsequent changes in seizure thresholds because the first baseline threshold measurements are known to drop drastically (but plateau to some extent) following the first stimulation (16).

### Diets Administration and ADT Measurements

Amygdaloid subjects were started on the control n-3 PUFA adequate diet or the experimental n-3 PUFA deficient diet, 1 week after the second baseline ADT measurement. The diets were mixed every 2–3 days in our laboratory, and stored at 4°C. The cornstarch and sucrose components of the diets were obtained from Disley Food Services (Scarborough, ON, Canada). The oils were obtained from Loblaws Supermarkets (Toronto, ON, Canada). Other ingredients (protein, fiber, vitamins, minerals and antioxidant) were obtained from Dyets Inc. (Bethlehem, PA, USA).

The control AIN-93G diet contained (g/kg): casein (200), cornstarch (530), sucrose (100), soybean oil (70), cellulose (50), vitamin mix (10), mineral mix (35), L-cysteine (3), choline bitartrate (2.5) and tertbutyl hydroquinone (0.014). The n-3 PUFA deficient diet contained identical macronutrient composition, but the source of fat was derived from 24 g/kg of safflower oil and 46 g/kg of coconut oil in lieu of the soybean oil. The fatty acid composition of the AIN-93G control and n-3 PUFA deficient diets is presented in Table 1.

**Table 1:** Fatty acid composition (% of total fatty acids) of the n-3 PUFA adequate and n-3 PUFA deficient diets.

	**n-3 PUFA adequate**	**n-3 PUFA deficient**
6:0	1.04 ± 1.80	0.72 ± 1.25
7:0	0.20 ± 0.22	0.16 ± 0.08
8:0	0 ± 0	7.96 ± 0.25
10:0	0.09 ± 0.08	5.56 ± 0.13
12:0	0.10 ± 0.09	35.04 ± 0.56
14:0	0.23 ± 0.01	11.71 ± 0.12
15:0	0.15 ± 0.01	0.11 ± 0.003
16:0	12.34 ± 0.23	8.23 ± 0.10
18:0	3.71 ± 0.12	2.24 ± 0.01
20:0	0.29 ± 0.01	0.12 ± 0.01
22:0	0.57 ± 0.32	0.18 ± 0.07
24:0	0.05 ± 0.08	0 ± 0
Total SFAs	18.77 ± 1.40	72.04 ± 0.36
18:1 t9	0 ± 0	0.092 ± 0.003
18:1 c9	16.28 ± 0.20	7.22 ± 0.08
18:1 c11	1.22 ± 0.02	0.31 ± 0.006
19:1 c7	0.297 ± 0.003	0 ± 0
22:1 n9	0 ± 0	0.07 ± 0.06
Total MUFAs	17.84 ± 0.25	7.70 ± 0.11
18:2 n6	53.58 ± 0.91	19.54 ± 0.26
18:3 n6	0.09 ± 0.08	0.12 ± 0.002
20:2 n6	0.18 ± 0.04	0 ± 0
Total n-6 PUFAs	53.85 ± 0.96	19.66 ± 0.27
18:3 n3	9.53 ± 0.21	0.60 ± 0.02
Total n-3 PUFAs	9.53 ± 0.21	0.60 ± 0.02

*Data are mean ± SD of n = 3 representative samples per diet. SFAs, saturated fatty acids; MUFAs, monounsaturated fatty acids; PUFAs, polyunsaturated fatty acids*.

### ADT Measurement Following DHA or Oleic Acid Administration

ADTs were measured between weeks 35–37 in the amygdaloid implanted subjects following OA or DHA injection (Figure 1A). The rats received a subcutaneous injection of DHA (600 mg/kg) or an equivalent dose of OA control 1 week after the last ADT was measured on week 34. A week later, treatments were switched, meaning that rats that received OA, received DHA. ADTs were measured following injection as described above. ADT measurements were initiated within 15 min post-injection. This ensured that each subject reached its expected ADT by ~1 h post-injection, in view of a study showing that it takes 1 h for DHA to increase seizure threshold (35).

The rationale for the 600 mg/kg dose is based on body weight. We had previously reported that DHA raises seizure thresholds in the PTZ seizure model 1 h following injection at a dose of 300–400 mg/kg in rats weighing 200–300 g (35–37). The rats in the present study weighed ~834 g at the time of seizure testing. Because unesterified DHA has a short plasma half-life and high volume of distribution associated with increased adiposity in heavier rats, the higher DHA dose of 600 mg/kg was selected to account for the greater body weight that likely increases the volume of distribution.

To compare the effects of OA and DHA on seizure thresholds, we subtracted the ADT measured on weeks 35 and 36, following OA or DHA injection, from the previous ADT on week 34, and the ADT following OA or DHA injection on week 37 from the ADT measured on weeks 35 and 36. In other words, the change in ADT was measured by subtracting the ADT following OA or DHA injection, from the prior ADT level.

### Dietary Treatment to Rats Used for Determining Brain Fatty Acid Composition

A parallel group of subjects were obtained from the breeding farm and placed on the n-3 PUFA adequate (*n* = 21) or deficient diets (*n* = 20) at the same time as the implanted animals (Figure 1B). They were handled in a similar manner upon arrival and throughout the course of the experiment, being placed in an open field for 30 min once a month. Two-thirds of these subjects were sacrificed at 16 and 32 weeks (*n* = 7 per group per time-point) post diet initiation with CO_2_ asphyxiation. The remaining one-third (*n* = 6–7 per group) was sacrificed at 33 weeks as described in the following section. The rationale for sacrificing the rats after 16 or 32 weeks of dietary treatment is based on the 4–12 weeks half-life of brain DHA, which led us to predict that a duration of 3–4 half-lives would be required to observe measurable effects of diet on brain DHA levels (31–33). The brains were excised immediately after CO_2_ asphyxiation, and dissected to separate piriform-amygdala from a 1 mm coronal section of the left hemisphere. The dissected pyriform-amygdala samples were stored in a minus 80°C freezer until they were subjected to fatty acid analysis as described below.

### PTZ Seizure Testing in Rats on the n-3 PUFA Adequate and Deficient Diets

The PTZ seizure test was performed on the remaining group of subjects with no implanted electrodes following 33 weeks of dietary treatment (Figure 1B; *n* = 14). Subjects were injected intraperitoneally with 110 mg/kg of PTZ, and observed in an open field for 10 min. This dose was chosen because it reliably induced tonic-clonic seizures in a separate group of subjects that were of the same age. The latency to the onset of myoclonic jerks and tonic-clonic seizures was determined by two observers, of whom one was blinded and the other was handling the animals. Subjects were euthanized with sodium pentobarbital (100 mg/kg) upon visibly showing tonic-clonic convulsions.

### Sacrifice and Tissue Fixation

Electrode-implanted subjects were deeply anesthetized with sodium pentobarbital (100 mg/kg), and subjected to a direct current of 100 μA for 30 s in order to lesion the site of the electrode implant for subsequent histological evaluation of the position of the electrode tip. The subjects were then decapitated, and the brains were dissected quickly and stored in formalin for a few weeks to ensure complete fixation of the tissue. The brains were then transferred to 20% sucrose solution containing 0.1% sodium azide and stored at 4°C for a few weeks until they were histologically examined.

### Histological Confirmation of Electrode Placement

The right hemisphere that contained the implanted electrode was used for histological confirmation as previously described. In brief, the hemispheres were chilled in isopentane on dry ice and sectioned using a cryostat (Leica Instruments, Willowdale, Ontario, Canada) maintained at −25°C. Coronal sections were obtained at a thickness of 40 μm and mounted onto gelatin coated glass slides. The electrode tract was visible to the naked eye, so sections were collected close to where the tract ended, and subsequently confirmed under light microscopy (Research Analysis System Model 421251; Amersham, MI). Subjects with misplaced electrodes were excluded from subsequent data analysis.

### Fatty Acid Analysis of the Pyriform-Amygdala

Total lipids were extracted from pyriform-amygdala by the method of Folch et al. (38) after being weighed to the nearest tenth of a milligram. The weighed samples were grinded in 6.5 ml of 0.9% KCl using a glass-grinder, and washed twice with 5 ml methanol, and twice with 10 ml of chloroform. Diheptadecanoyl L-α-phosphatidylcholine (Sigma, St. Louis, MO) in chloroform was added as an internal standard to the total lipid extracts, which were then dried under nitrogen and reconstituted in 2 ml of chloroform.

Total lipids directly methylated in 14% methanolic BF_3_ (2 mL) and hexane (2 ml) at 100°C for 1 h. The samples were allowed to cool at room temperature for 10 min and centrifuged at 1,200 g following the addition of deionized water (2 ml). The upper hexane layer containing fatty acid metyl esters (FAMEs) was reconstituted in 50 μl hexane and analyzed by gas-chromatography as described in the following section.

### Fatty Acid Methyl Ester Analysis by Gas-Chromatography

FAMEs were analyzed on a Varian-430 gas chromatograph (Varian, Lake Forest, CA, USA) equipped with a Varian FactorFour capillary column (VF-23 ms; 30 m × 0.25 mm i.d. × 0.25 μm film thickness) and a flame-ionization detector. FAMEs were injected in splitless mode. The carrier gas was helium, set to a constant flow rate of 0.7 ml/min. The injector and detector ports were set at 250°C. FAMEs were eluted using a temperature program set initially at 50°C for 2 min, increased at 20°C/min to 170°C held at 170°C for 1 min, increased at 3°C/min to 212°C and held at 212°C for 5 min. Peaks were identified by retention times of authentic FAME standards of known composition (Nu-Chek-Prep, Elysian, MN).

### Dietary Fatty Acid Analysis

The fatty acid composition of the n-3 PUFA adequate and deficient diets was also determined by gas-chromatography. Total lipids were first extracted from ~0.5 g of powdered diet in chloroform/methanol (2:1 v/v) after adding 2 mg of unesterified heptadecanoic acid as an internal standard (Sigma, St. Louis, MO). Saline (0.9% w/v, 2 ml) was added to separate polar compounds. The bottom layer containing total lipids was transferred to test-tubes. A portion of the extract was dried under nitrogen, reconstituted in 2 ml of hexane, and directly methylated with 2 ml of 14% boron trifluoride in methanol at 100°C for 1 h. The hexane layer was extracted and FAMEs were analyzed by gas-chromatography as described above.

### Data Presentation and Statistical Analysis

The data are presented as means ± SD. Data analysis was performed using Sigma Stat v.3.2 (Jandel Corporation) or Graphpad Prism v 5.0 (La Jolla, CA). A two-way repeated measures analysis of variance (ANOVA) was used to determine the effects of diet and time on body weight, ADT, seizure duration and seizure score. A two-way ANOVA was used to test the effect of diet and time on pyriform-amygdala DHA and n-6 DPA composition. Due to the small sample size, the Mann-Whitney *U*-test was used to assess differences in the latency to PTZ-induced seizures between the n-3 PUFA adequate and deficient groups. Statistical significance was accepted at *P* < 0.05.

## Results

### Electrode Placements

Although, the surgeries were aimed at placing the electrodes within the basolateral amygdala, subjects with electrodes falling within the amygdaloid complex were included in the analysis. Electrodes were successfully implanted within the amygdala in 5 out of 8 n-3 PUFA adequate control rats and 4 out of 8 n-3 PUFA deficient rats. Subjects with electrodes misimplanted outside the amygdaloid complex were, therefore, excluded from subsequent analyses.

In the successfully implanted subjects, electrodes were within the basolateral amygdala for 3 (out of 5) and 2 (out of 4) subjects within the n-3 PUFA adequate and deficient groups, respectively. The electrodes for the remaining subjects were within the amygdala, but slightly medial or anterior to the basolateral amygdala. We were not able to compare differences in ADTs within the different amygdala foci due to the small sample size, which is why we accepted subjects with successful implants anywhere within the amygdala. This is also consistent with our primary hypothesis, which aimed to test the effects of diet on amygdaloid seizure thresholds.

### Body Weight

**Figure 2:**
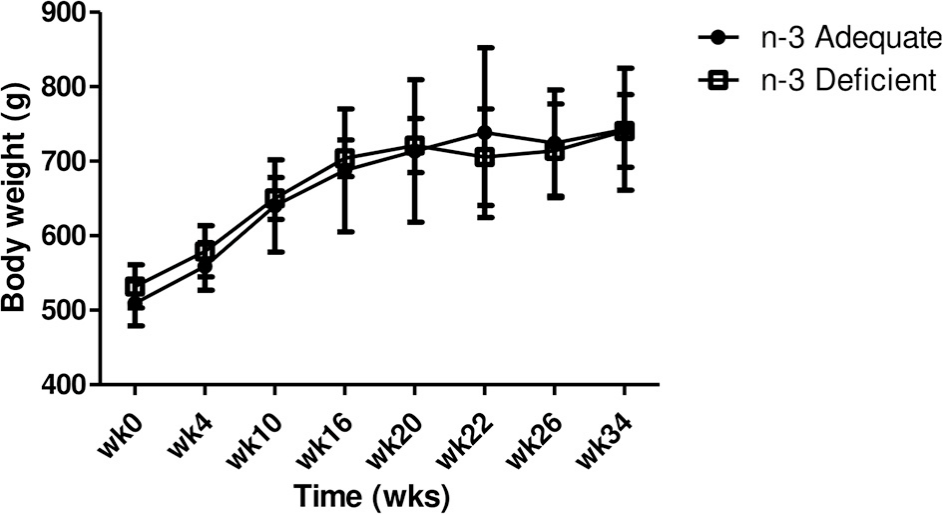
Effects of chronic n-3 PUFA deficiency on body weight over time. Rats were placed on a n-3 PUFA adequate (control) or n-3 PUFA deficient diet. Data are mean ± SD of *n* = 5 n-3 PUFA adequate controls and 4 n-3 PUFA deficient rats. Two-way repeated measures ANOVA showed a significant effect of time on body weight (*P* < 0.0001). There was no significant effect of diet on body weight (*P* > 0.05). Subjects' weights increased over time, regardless of diet.

The data related to body weight of implanted rats from the start of the baseline ADT measurements and throughout the 34 weeks period of threshold measurements is shown in Figure 2. A two-way repeated measured ANOVA showed a significant effect of time (*P* < 0.0001) but not of dietary treatment (*P* > 0.05) on body weight. As shown in Figure 2, both n-3 PUFA adequate and deficient rats gained weight over time. There were no significant differences in body weight between the groups at any time point.

### Effect of Dietary n-3 PUFA on Amygdaloid ADT and Seizure Duration

Seizures were successfully recorded at baseline (“week 0”) and at 4, 10, 16, 20, 22, 26 and 34 weeks thereafter. Recordings were obtained for all subjects with amygdala implants, except for one n-3 PUFA adequate control rat on weeks 22, 26, and 34 and one n-3 deficient rat on weeks 10, 16, and 20. These two subjects did not show an afterdischarge when measured during these time periods, probably due to a transient (but unconfirmed) infection. We were not able to retrieve raw ADT files for one control rat on week 16 and one n-3 deficient rat on week 20. ADTs that were not successfully obtained for these 4 rats during the 1–3 time-points were not included in the statistical analysis. Thus, ADTs successfully recorded for these 4 rats during other weeks, as well as the rest of the amygdala-implanted subjects, were included in the statistical analysis.

**Figure 3:**
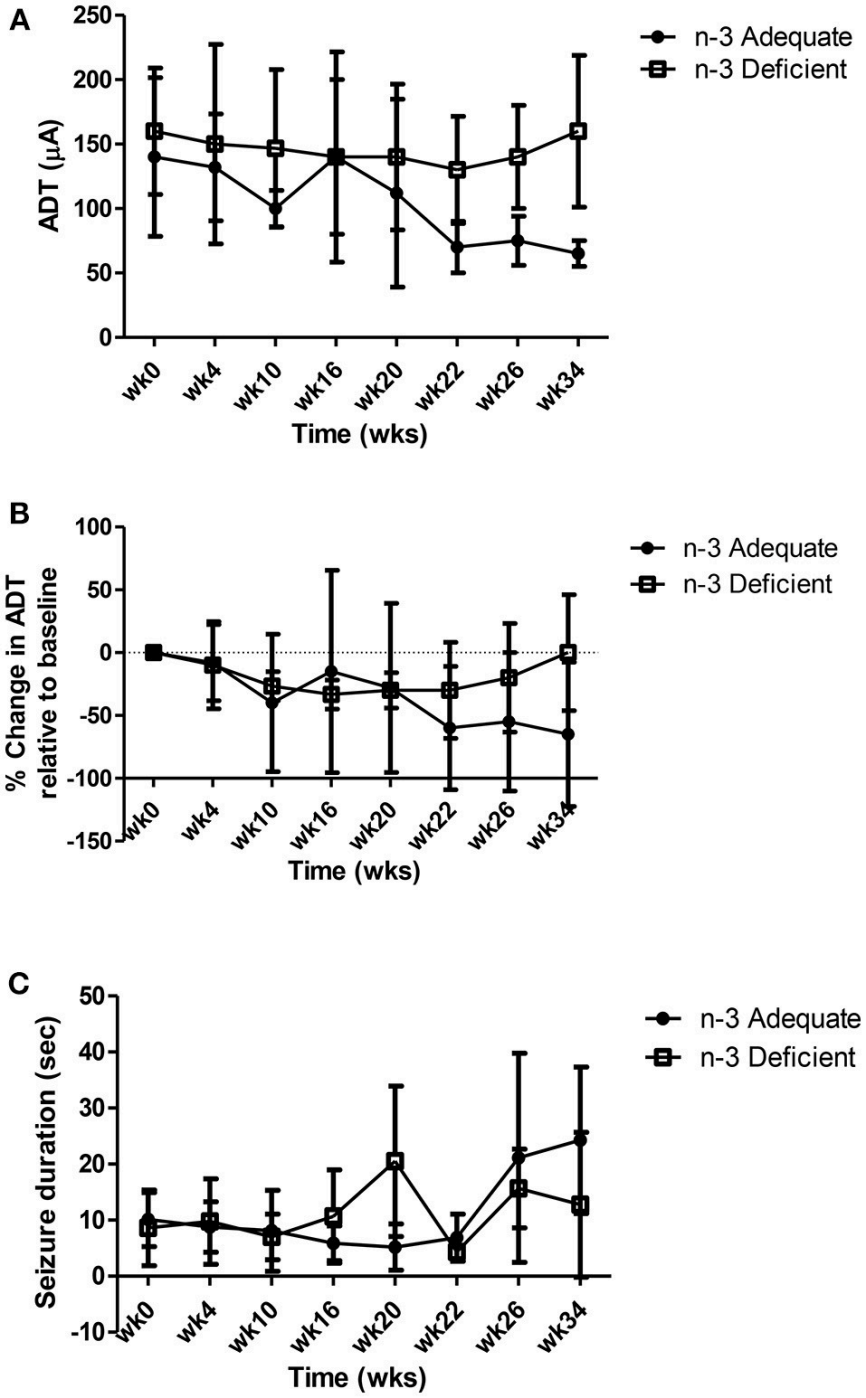
Effect of chronic n-3 PUFA deficiency on **(A)** ADT (μA), **(B)** percent change in ADT over time and **(C)** seizure duration. ADTs and seizure duration were recorded for 34 weeks from rats maintained on the n-3 PUFA adequate diet (*n* = 5) or n-3 PUFA deficient diet (*n* = 4). Two-way repeated measures ANOVA showed a significant effect of time but no effect of treatment or interaction between time and treatment on ADT, % change in ADT and seizure duration. ADT **(A)** and the % change in ADT **(B)** decreased gradually over time in both n-3 adequate and deficient rats, whereas seizure duration increased over time **(C)**.

The data related to ADT (μA), percent change in ADT from baseline and seizure duration over the 34 week measuring period are presented in Figures 3A–C. Two-way repeated measures ANOVA showed a significant effect of time but no effect of treatment or interaction between time and treatment for ADT, % change in ADT and seizure duration. As shown in Figure 3, ADT (Figure 3A) and the % change in ADT (Figure 3B) decreased gradually over time in both n-3 adequate and deficient rats, whereas seizure duration increased over time (Figure 3C). A peculiar observation is that absolute ADT values appeared to decrease more for the n-3 PUFA adequate group at 26 and 34 weeks (Figure 3A), but after correcting for the small but insignificant differences in baseline ADT between the two groups, this effect was no longer seen (Figure 3B).

### ADT Following OA or DHA Injection

OA or DHA (600 mg/kg) were injected subcutaneously to n-3 PUFA adequate and deficient rats between weeks 35 and 37, to test whether DHA raises ADT. In particular, we wanted to address whether ADTs increased in n-3 deficient rats following DHA injection.

**Figure 4:**
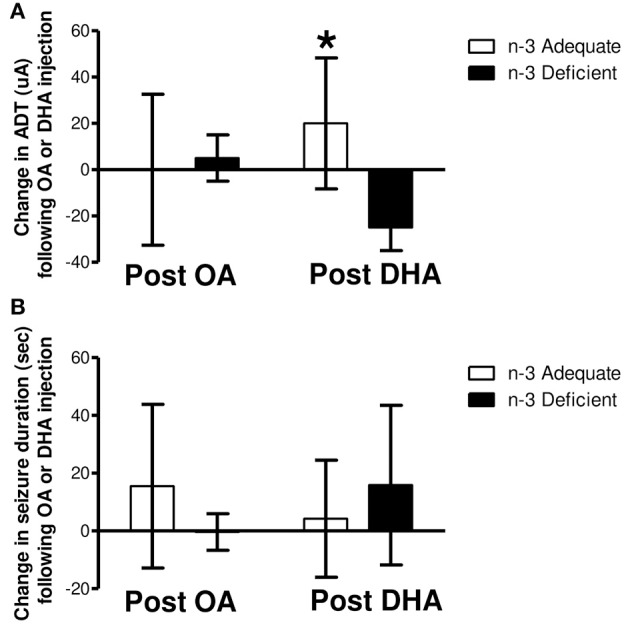
Change in ADT 1 h post s.c. injection of OA or DHA (600 mg/kg) to rats fed an n-3 PUFA adequate or deficient for 37 weeks. Rats of each dietary group randomly received 600 mg/kg of oleic acid (OA) or docosahexaenoic acid (DHA) approximately 1 week after the last ADT was taken on week 34. ADTs and seizure duration were measured within 1 h of fatty acid treatment. One week later, the treatments were switched (i.e. rats that received OA now received DHA), and ADTs were measured within 1 h of fatty acid treatment. The difference in ADT following OA and DHA treatment of each subject, per diet, was determined (i.e., DHA ADT—OA ADT) using the prior ADT as a reference point. As shown in **(A)**, two-way ANOVA revealed no main effect of diet or fatty acid treatment on the change in ADT. A significant diet and fatty acid treatment interaction was detected (*P* < 0.05). *Post hoc* analysis of the means with Bonferroni's *post-hoc* test indicated that the change in ADT following DHA treatment was significantly greater in rats on the n-3 adequate diet as compared to rats on the n-3 PUFA deficient diet (^*^P < 0.05). No significant main effects or interaction were detected for seizure duration **(B)**.

Figure 4 shows the change in ADT and seizure duration following s.c., injection of n-3 PUFA adequate and deficient rats with OA or DHA. Two-way ANOVA revealed a significant interaction between diet and fatty acid injection (*P* = 0.048), but no significant main effect of diet or injection on ADT (Figure 4A). *Post-hoc* analysis with Bonferroni's *post-hoc* test indicated that the change in ADT following DHA treatment was significantly higher in rats on the n-3 adequate group as compared to rats on the n-3 deficient group (*P* < 0.05). The change in ADT following OA injection was not significant between n-3 adequate or deficient rats. Also, no significant differences between OA and DHA injection were observed.

Two-way ANOVA showed no significant effect of diet or fatty acid (OA or DHA) injection on seizure duration (Figure 4B). Also, no significant interaction was detected.

### Latency to PTZ-Induced Seizure Onset Following 33 Weeks of Treatment With an n-3 PUFA Adequate or Deficient Diet

Body weights measured at the time of seizure testing were not significantly different between the two groups (n-3 PUFA adequate, 873 ± 125 g, *n* = 7; n-3 PUFA deficient 789 ± 116 g, *n* = 6, *P* = 0.29 Mann-Whitney *U*-test).

**Figure 5:**
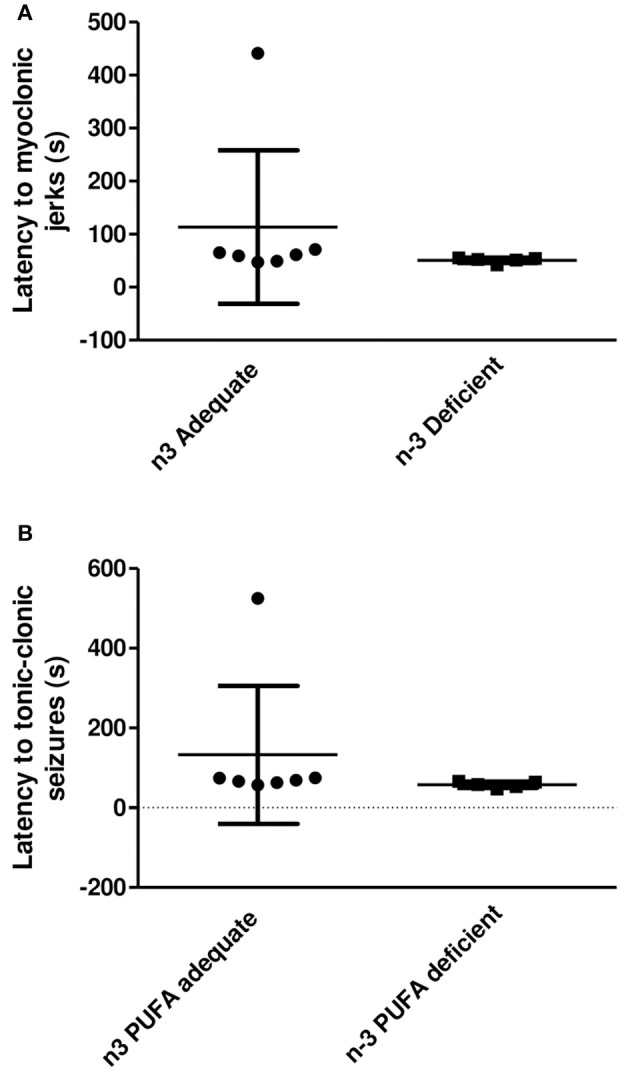
Latency to the onset of myoclonic jerks and tonic clonic seizures following PTZ administration (110 mg/kg, i.p.) to rats treated with an n-3 PUFA adequate or deficient diet for 33 weeks. One rat from the n-3 PUFA deficient group was excluded because it did not seize within the 10 min observation period. Data are mean ± SD of *n* = 7 n-3 PUFA adequate and *n* = 5 n-3 PUFA deficient subjects. **(A)** Latency to the onset of myoclonic jerks. A Mann-Whitney U was used to compare latencies. The latency to the onset of myoclonic jerks did not differ significantly between the groups (*P* = 0.15). **(B)** Latency to the onset of tonic-clonic seizures. A Mann-Whitney U was used to compare latencies. The latency to the onset of tonic-clonic seizures was lower in the n-3 deficient group as compared to the adequate group. This difference, however, was not statistically significant (*p* = 0.06).

The data related to the onset of myoclonic jerks and tonic-clonic seizures following PTZ administration to non-implanted rats maintained on the n-3 PUFA adequate or deficient diet are presented in Figure 5. One rat from the PUFA n-3 deficient group did not seize within the 10 min observation period so it was not included in the statistical analysis. As shown in Figure 5A, the latency to mycolonic jerks in the rats that seized did not differ significantly between the groups (*P* = 0.15 by Mann-Whitney *U*-test). The latency to tonic-clonic seizures (Figure 5B) was lower by 57% in the n-3 PUFA deficient group as compared to controls, but the difference was not statistically significant (*P* = 0.06).

### Piriform-Amygdala DHA and N-6 DPA Composition

**Figure 6:**
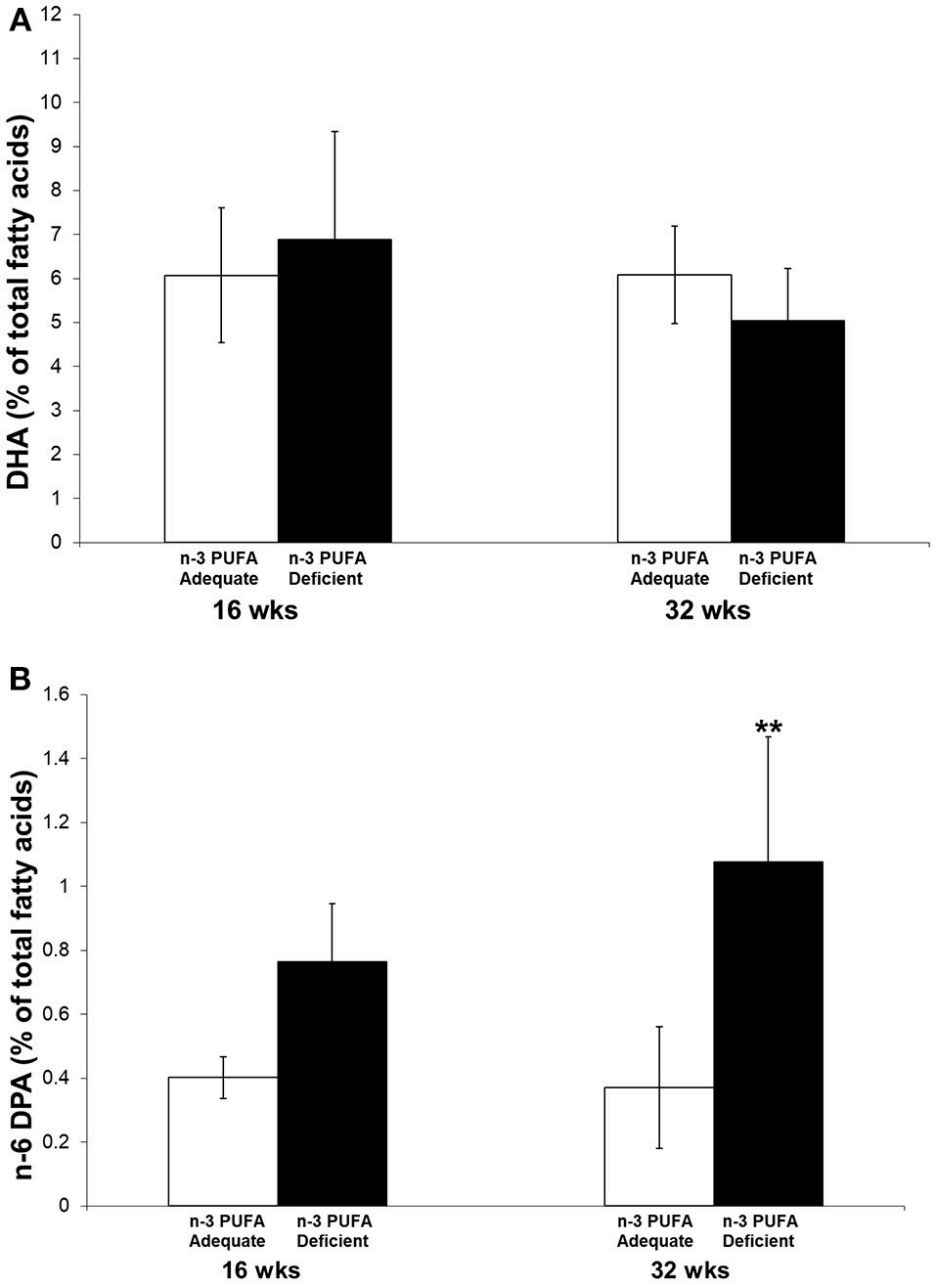
DHA **(A)** and n-6 DPA **(B)** % of total fatty acids in piriform-amygdala of rats fed an n-3 PUFA adequate or deficient diet for 16 and 32 weeks. Data are mean ± SD of *n* = 7 per diet group per time-point for each fatty acid, except n-6 DPA, for which *n* = 6 in control rats at 32 weeks because it was not detected in one sample. A two-way ANOVA followed by Benferroni's *post-hoc* was used to assess the effect of diet and time on pyriform-amygdala DHA and n-6 DPA % composition. There was no significant effect of diet, time or interaction between diet and time on DHA percent composition. There was a significant effect of time (*P* = 0.0261) and diet (*P* = 0.0006) on n-6 DPA percent composition, but no significant interaction was detected (*P* = 0.0997). *Post-hoc* comparison of the means indicated that the difference between the n-3 adequate and deficient group was significant at 32 weeks (^**^P < 0.01).

Piriform-amygdala DHA and n-6 DPA were measured in a parallel group of non-electrode implanted subjects administered the n-3 PUFA adequate or deficient diets for 16 and 32 weeks. Figure 6 shows piriform-amygdala DHA (6-A) and n-6 DPA (6-B) composition, expressed as percentage of total fatty, of rats fed an n-3 PUFA adequate or deficient diet for 16 and 32 weeks. A two-way ANOVA followed by Benferroni's *post-hoc* was used to assess the effect of diet and time on pyriform-amygdala DHA and n-6 DPA% composition.

There was no significant effect of diet, time or interaction between diet and time on DHA percent composition. There was a significant effect of time (*P* = 0.0261) and diet (*P* = 0.0006) on n-6 DPA percent composition, but no significant interaction was detected (*P* = 0.0997). *Post-hoc* comparison of the means indicated that the difference between the n-3 adequate and deficient groups at 32 months was statistically significant. N-6 DPA was 2.9-fold higher in the n-3 PUFA deficient group as compared to the n-3 PUFA adequate group.

## Discussion

The present study showed that chronic dietary n-3 PUFA deficiency, achieved by removing ALA from the diet, did not significantly alter amygdaloid seizure thresholds or latency to PTZ-induced seizures. Acute OA injection did not alter ADTs, whereas DHA increased amygdaloid ADTs in the n-3 PUFA adequate group relative to the n-3 PUFA deficient group. Chronic PUFA n-3 deficiency increased pyriform-amygdala n-6 DPA percent composition without altering DHA composition.

Our findings do not support the hypothesis that n-3 PUFA deficiency lowers seizure thresholds in adult rats. This may be related to the fact that the amygdala-pyriform DHA composition was not reduced following dietary n-3 PUFA deprivation.

Previous studies reported a significant reduction in cortical and whole brain DHA concentrations and percent composition following chronic n-3 PUFA deficiency (39–41). The lack of changes in DHA composition in the pyriform-amygdala suggests that this brain region may be less sensitive to the effects of dietary n-3 PUFA manipulation as compared to other brain regions. Consistent with this suggestion, we reported that chronic fish oil supplementation to rats for 6 months did not significantly increase piriform-amygdala DHA concentration (16).

Another possibility accounting for the lack of change in piriform-amygdala DHA composition is that the n-3 PUFA deficient diet was initiated during adulthood (at 5 months of age). Other studies initiated n-3 PUFA deficiency during development or at weaning (~21 days post-partum) (39–41). In rats, DHA accretes in the brain during the first 29 days of life, and early n-3 PUFA deficiency interferes with brain DHA accretion and concentration (42). The extent of brain DHA depletion when dietary n-3 PUFA deficiency is initiated during adulthood, and after DHA accretion rate has reached steady-state, is not known. It is possible that adipose tissue contributes to maintaining pyriform-amygdala DHA status throughout adulthood when n-3 fatty acids are absent from the diet (43). An alternative but unconfirmed hypothesis is that DHA turnover within the pyriform-amygdala complex is slow compared to other brain regions such as the cortex. Regional differences in brain DHA turnover in relation to concentration should be further examined in future studies.

N-6 DPA, a marker of n-3 PUFA deficiency was significantly increased 32 weeks after the rats were started on the n-3 PUFA deficient diet. It is unlikely that n-6 DPA altered ADTs, because it was reported to have no effect on excitatory hippocampal sharp waves *ex vivo* (44). Future studies should confirm these findings *in vivo*, however.

Pages et al. reported that mice fed an n-3 PUFA deficient diet for 30–34 days were more susceptible to magnesium-dependent audiogenic seizures than mice fed an n-3 PUFA adequate diet (15, 26). We did not observe significant changes in amydgaloid afterdischarge or PTZ seizure thresholds following n-3 PUFA deprivation in the present study. Differences in study outcomes are likely due to the seizure model used. The studies by Pages et al. used a magnesium deficient diet, which may have lowered seizure thresholds sufficiently for audiogenic provocation (15, 26).

Acute DHA injection increased amygdaloid seizure thresholds in rats on the n-3 PUFA adequate diet, but not in rats on the n-3 PUFA deficient diet, while OA had no significant effect on ADTs. The increase in amygdaloid seizure thresholds following DHA injection in the n-3 adequate group is consistent with previous studies which showed that injected or dietary DHA raises seizure thresholds in rats on an n-3 adequate diet (16, 17, 36, 37, 45, 46).

In our previous studies, the increase in seizure latency following acute DHA injection was attributed to the increase in plasma unesterified DHA concentration (37), the form available for brain uptake (32, 47, 48). The lack of significant effect of unesterified DHA on amygdaloid ADTs in the n-3 PUFA deficient group, is likely because injected DHA did not increase plasma unesterified DHA concentrations to therapeutic levels. Plasma unesterified DHA before and after acute DHA administration to n-3 PUFA adequate and deficient rats was not measured in the present study, a limitation which should be addressed in future studies. Unesterified DHA is known to reduce neuronal excitability by acting on GABA or voltage gated ion channels (44, 49–51), or through its oxygenated metabolites such as neuroprotectin D1 (21), which were reported to reduce electrically induced hippocampal excitability in rodents (52).

The North American diet may be low in DHA (53–55), but it is not omega-3 deficient *per se*. Although extreme n-3 deficiency as modeled in the present study is not likely to be clinically prevalent (53–55), this study demonstrates the importance of dietary n-3 PUFA status as a potential modifier of the anti-seizure effects of DHA. It is not clear, however, as to whether people with epilepsy have low or deficient n-3 fatty acid intake or circulating DHA levels.

The main limitation of this study is the low sample size. While the repeated stimulations over time confirm no changes in ADT between the diets, the PTZ and acute OA and DHA injection experiments were only performed once. These studies should be reproduced with a larger number of subjects. Another limitation is that not all electrodes were within the intended basolateral amygdala target; some were medial or lateral but were within the amygdala. Thus, our findings cannot be generalized to the basolateral amygdala or specific structures within.

In conclusion, dietary n-3 PUFA deprivation for 8–9 months did not alter amygdaloid seizure thresholds or the latency to PTZ-induced seizures. Injected DHA, however, raised amygdaloid seizure thresholds in rats on the n-3 PUFA adequate diet, but had no effect in rats on the n-3 PUFA deficient diet, suggesting that dietary n-3 PUFA status may modulate the anti-seizure effects of DHA. Clinical assessment of dietary n-3 fatty acid background and circulating DHA status is warranted in intervention studies addressing the role of DHA supplementation in people with epilepsy. Understanding the role of DHA in raising seizure thresholds may reduce seizure incidence and the risk of SUDEP in at-risk individuals (56–58).

## Author Contributions

AT, RB, and WB designed the study. AT and M-OT contributed to the data and statistical analysis. AT, M-OT, AS, NT, FC, and MJ performed the experiments.

### Conflict of Interest Statement

The authors declare that the research was conducted in the absence of any commercial or financial relationships that could be construed as a potential conflict of interest. The handling Editor declared a past co-authorship with one of the authors AT.
